# Coverage Root after Removing Peripheral Ossifying Fibroma: 5-Year Follow-Up Case Report

**DOI:** 10.1155/2016/6874235

**Published:** 2016-11-07

**Authors:** Paulo S. G. Henriques, Luciana S. Okajima, Marcelo P. Nunes, Victor A. M. Montalli

**Affiliations:** ^1^Department of Periodontology, São Leopoldo Mandic Institute and Research Center, Campinas, SP, Brazil; ^2^Department of Oral Pathology, São Leopoldo Mandic Institute and Research Center, Campinas, SP, Brazil

## Abstract

When lesions in soft tissue reach the gingival margin, they can produce aesthetic defects during its permanence and after its removal. Periodontal plastic surgery allows the correction of the gingival contour using different techniques. This paper is a case report of a peripheral ossifying fibroma removal in the interproximal area of teeth 21 and 22 in addition to root coverage of the affected area through two surgical phases: keratinized gingival tissue augmentation surgery with free gingival graft concurrent with removal of the lesion and, in a second stage, root coverage by performing coronally advanced flap technique with a follow-up of five years. The initial results achieved, which were root coverage of 100% after 6 months, promoted an adequate gingival contour and prevented the development of a mucogingival defect or a root exposure with its functional and aesthetic consequences. After five years, the results showed long term success of the techniques, where the margin remained stable with complete root coverage and tissues were stable and harmonic in color.

## 1. Introduction

Peripheral ossifying fibroma is characterized as a hyperplasic gingival mass with calcified foci, supposedly formed by metaplastic bone [1]. The bone is found in the middle of a nonencapsulated proliferation of bulky benign fibroblasts. The lesion may be derived from the connective tissue of the submucosa or the periodontal ligament. There is a tendency for the presence of inflammatory cells in the outer portion of the lesion. The surface often shows ulcerated areas and rarely causes erosion of adjacent bone [2].

The peripheral ossifying fibroma, also known as ossifying fibroid epulis, ossifying fibroma with calcification, peripheral cement-ossifying fibroma, and calcifying fibroblastic granuloma, is also part of the nonneoplastic proliferative lesions [3].

It is considered a reactive lesion, although its pathogenesis is uncertain. This pathology appears as a tissue response to chronic long term stimulation. This can occur when the gum tissue reacts in response to irritants such as biofilm and subgingival calculus, misplaced teeth, restorations over contour, ill-fitting dentures, root remnants, poorly preserved teeth, foreign bodies in the gingival sulcus, and orthodontic treatment. There is a mesenchymal cell of the periodontal ligament and/or cementum proliferation that are induced by such local irritants. The displacement and mobility of the teeth are uncommon, unless preexisting periodontal disease is found or in cases where the teeth are erupting [4].

Clinically, it appears as a nodular lesion, exophytic, pedunculated in most cases, of streaky reddish coloration of whitish areas, or similar in color to the adjacent mucosa. It features bright and opaque surface in some spots and irregular texture and contours, with slow growth rate, although it is able to reach large dimensions [5].

This injury is located, preferably, in the attached gingiva or exceptionally in the free marginal gingiva. There is a predilection for the anterior portion of the jaws [4, 5]. Sometimes it extends throughout the teeth, involving both the facial and the lingual gum [4]. There may be bleeding when the lesion is touched or even spontaneously, but mainly when it is constantly traumatized. In most cases the patient is asymptomatic [3, 5].

Women are affected more often than men by this injury, which occurs predominantly in the second decade of life [4, 5] and in Caucasians [3, 5], accounting for 9.6% of gingival lesions [6].

Other injuries that have similar clinical appearance to peripheral ossifying fibroma include pyogenic granuloma, peripheral giant cells granuloma, fibrous hyperplasia, and giant cell fibroma [1, 5]. All these injuries are caused by low intensity chronic irritation.

The treatment of choice is local excision, which should include the periodontal ligament, if it is also involved. Furthermore, one should remove any identifiable causative agent [1, 4, 6]. There may be recurrence [4, 6], but its risk is diminished if the excision is performed under the periosteum [2, 6].

The literature provides several ways of removing the lesion, such as the use of Nd:YAG laser or conventional surgery with scalpel [7].

The excisional biopsy necessary for this case is aggressive and may result in a severe periodontal defect because it can involve the entire keratinized adjacent tissue creating a similar Class I or II Miller defect. When trying to recreate the excised tissue, several approaches can effectively increase the present tissue, such as a graft of the subepithelial connective tissue, free gingival graft, derivatives of the enamel matrix, guided tissue regeneration, and coronal or lateral advanced flaps. The choice of technique will depend on the amount of tissue to be recreated [8].

## 2. Case Report

**Figure 1:**
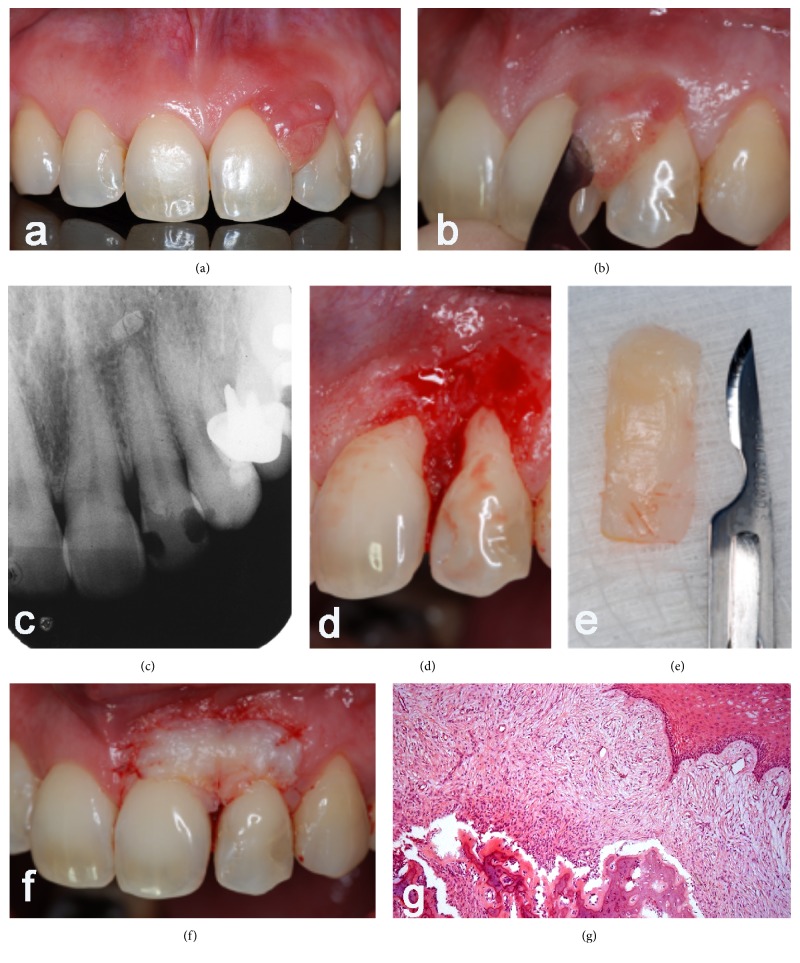
(a) Intraoral physical examination showed an injury inserted in the interproximal gum; ((b), (d), and (e)) excision of the lesion; (c) radiographic examination with no changes observed. (g) Histological diagnosis of peripheral ossifying fibroma.

A 36-year-old female leucoderma patient sought treatment complaining of a lesion located between teeth 21 and 22, painless and compromising the aesthetics of her smile (Figure 1(a)). Intraoral physical examination showed an injury inserted in the interproximal gum, measuring 1.2 × 0.9 × 0.5 cm on the facial surface and 0.7 × 0.5 × 0.3 cm in the palatal face, exophytic and nodular. The radiographic examination showed no related changes (Figure 1(c)).

Surgical techniques were performed as described below: after local anesthesia with 2% lidocaine with epinephrine at a concentration of 1 : 100,000, the excision of the lesion was proceeded with a 15C scalpel blade (Figures 1(b) and 1(e)), removing all the gingival and periodontal tissue involved, followed by scaling and root planing of the same teeth (Figure 1(d)).

After excision of the lesion, the removal of a free gingival graft from the palate was performed, which was placed in the exposed conjunctive tissue area to recreate the band of keratinized tissue lost as a result of the lesion itself and its excision. The graft was taken from the palate and its format was similar to the open area of the receiving tissue (Figure 1(f)). The apical and coronal dimension and thickness were measured so that it could be suitable and uniform. The graft was sutured along its entire length (Figure 1(f)). Digital pressure was performed with saline moistened gauze to remove any blood clot and maintain the graft in intimate contact with the recipient bed.

The material obtained from excisional biopsy was sent for pathological analysis. Histologically, the lesion showed an intact squamous epithelium and in the lamina propria a highly cellular component of fibroblasts was observed with central area of calcification, setting the diagnosis for peripheral ossifying fibroma (Figure 1(g)).

**Figure 2:**
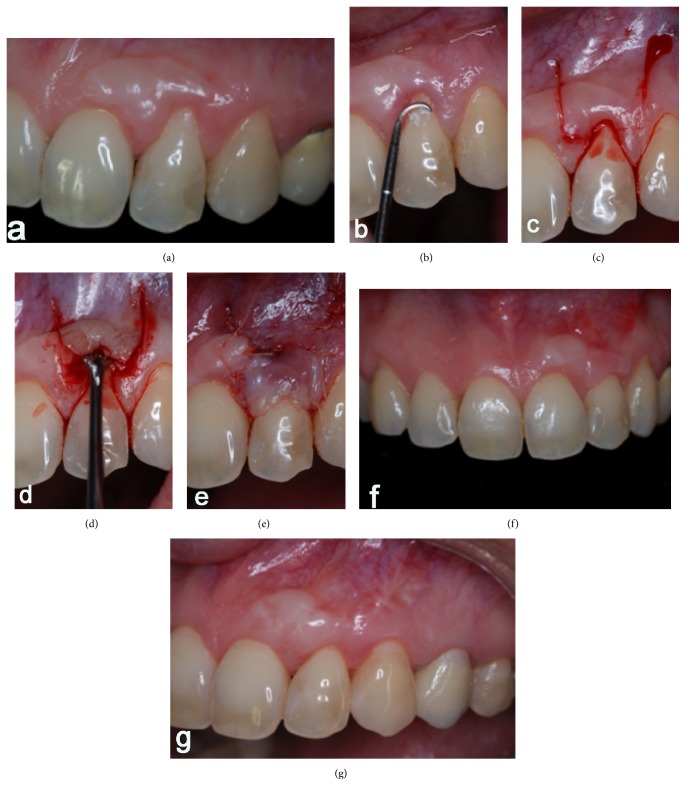
(a) After three months of the procedure; ((b), (c), (d), and (e)) a second surgical stage was performed in order to cover the exposed root of tooth 22; (f) root coverage of 100% after 6 months and (g) 5-year follow-up.

Three months after the procedure (Figure 2(a)), a second surgical procedure was performed in order to cover the exposed root of tooth 22. The biomechanical preparation of the surface of the root was accomplished with scaling and root planing (Figure 2(b)) and application of EDTA 24% neutral pH (Pref-Gel®, Straumann). The coronally advanced flap technique, described by de Sanctis and Zucchelli (2007) [9], was the selected technique: two horizontal beveled incisions were performed, mesial and distal to the recession, located at one end of the anatomical papillae and equal to the height of the recession plus 1 mm; two oblique incisions, slightly divergent, starting at the end of the two horizontal incisions and extending to the alveolar mucosa (Figure 2(c)). The coronal portion of the flap is partially divided, while the portion apical to the recession is a full thickness flap, exposing 3-4 mm of bone (Figure 2(d)). The relaxing vertical incisions are elevated in partial thickness. Apical bone exposure is held in the partial thickness flap, ending where it is possible to passively move the flap in coronal direction and coronally in the cementum-enamel junction. At this time simple sutures are performed throughout the flap (Figure 2(e)).

After the initial results were achieved, root coverage of 100% was obtained after 6 months (Figure 2(f)), and suitable gingival contour was promoted which prevented the development of a mucogingival defect or root exposure with its functional and aesthetic consequences. After five years (Figure 2(g)) the margin remained at its initial position, with no relapse in the exhibition of the cementum-enamel junction; and tissues were stable and characterized by color harmony, demonstrating the success of the chosen techniques.

## 3. Discussion

The gingiva when subjected to local chronic irritation or trauma reacts with localized hyperplasia that can be composed of mature collagen, cellular fibroblastic tissue, mineralized tissue, endothelial tissue, and multinucleated giant cells (3). Clinical and histological examinations are essential to achieve a diagnosis and ensure a complete treatment plan, which, in this case, included not only the removal of the lesion, but also reconstruction of the anterior esthetic zone impaired when performing the biopsy.

5-year follow-up of this case showed no recurrence of the lesion. Our findings are in accordance with Silva et al. (2007) [10], who presented a case report of a surgical excision of a peripheral ossifying fibroma coincident with central odontogenic fibroma with an uneventful follow-up of one year.

Excisional biopsies when performed frequently result in mucogingival defects, which may produce esthetic problems and increase the chance of hyperesthesia [11].

Bernimoulin et al. (1975) [12] first described a root coverage technique with free gingival graft placed to increase the zone of keratinized gingiva and flap coronally repositioned later.

Besides having an important role in maintaining gingival health, the attached gingiva protects the periodontium against external injuries, maintains a stable position of the gingival margin, and dispels the physiological forces made by the muscle fibers of the alveolar mucosa against the gum tissues. There is controversy regarding the amount of keratinized tissue to maintain gingival health. Mucogingival techniques are present in the literature to increase the zone of attached gingiva. Among the alternatives, the free gingival graft is a widespread procedure, because of abundant donor site and the possibility of treating multiple teeth. As disadvantages we can cite postoperative discomfort, unpredictable color harmony, and the need for a second donor site [13].

During this treatment, biopsy and the free gingival graft were performed at the same surgical procedure. This decision was made to avoid repetitious postoperative discomfort for the patient and to make oral hygiene procedures more effective in accordance with Anderegg and Metzler (1996) [14] and Keskiner et al. (2016) [15].

The decision of performing first the free gingival graft found evidence in literature which points out that thin adjacent gingiva makes root coverage less predictable [16] and an adequate amount of attached gingiva improves periodontal health [17]. A systematic review [18] stated that the free gingival graft is a successful treatment concept to increase the width of attached gingiva around teeth. In this case report, as described also by other authors [9], clinical increases in the apicocoronal dimensions of keratinized tissue and attached gingiva were observed.

Root exposure, as a side effect of the biopsy, can be corrected after the recreation of keratinized tissue band. The coronally advanced flap technique (CAF) is a great alternative treatment because it presents satisfactory results in long term root coverage, good color harmony of the area treated with the surrounding tissues [19], without an excessive increase in the thickness of the tissue, and complete recovery of the original morphology of the marginal soft tissue. It also presents better postoperative course when compared to coronally advanced flap with connective tissue graft [19]. The only limiting factor to this technique is the need of a band of at least 1 mm keratinized tissue [11]. A systematic review performed in 2008 [20] confirmed that the coronally advanced flap procedure is a safe and reliable approach in periodontal plastic surgery and is associated with consistent recession reduction and frequently with complete root coverage.

Coronally advanced flap can be associated with different materials as membrane barriers [21, 22], grafts, subepithelial connective tissue [19], porcine collagen matrix [23, 24], platelet-rich plasma [25, 26] and platelet-rich fibrin [27, 28].

Consensus Report of the European Workshop on Periodontology in 2014 [29] claimed that periodontal plastic procedures are complex, technique-sensitive interventions that require advanced skills and expertise. The choice of the technique should take in account increased morbidity when having a donor area or increased cost when using allograft materials. When there is enough tissue in the area to provide a well-designed flap for root coverage with stability, there is no need to use a soft tissue graft.

The treatment for gingival recession is considered completely successful when root coverage is associated with a gingival margin and a crevice probing depth that is coronal to the cementoenamel junction [30], as presented in this case report with 5-year follow-up.

## 4. Conclusion

Peripheral ossifying fibroma is a benign, slowly progressive lesion, with limited growth and histopathologic confirmation is mandatory. Complete surgical excision down to the periosteum is the preferred treatment and close postoperative follow-up is required. Surgical procedures with two stages using free gingival graft and coronally advanced flap present good results. In the presence of sufficient keratinized tissue, coronally advanced flap shows efficacy in root coverage.
